# Association between nocturnal hypoxia and liver injury in the setting of nonalcoholic fatty liver disease

**DOI:** 10.1007/s11325-014-1008-7

**Published:** 2014-05-29

**Authors:** Qi-Chang Lin, Li-Da Chen, Gong-Ping Chen, Jian-Ming Zhao, Xiao Chen, Jie-Feng Huang, Li-Hua Wu

**Affiliations:** 1 Fujian Provincial Sleep-Disordered Breathing Clinic Center, Fuzhou, Fujian Province People’s Republic of China; 2 Laboratory of Respiratory Disease of the Fujian Medical University, Fuzhou, Fujian Province People’s Republic of China; 3 Department of Respiratory Medicine, The First Affiliated Hospital of Fujian Medical University, No, 20, Chazhong Road, Taijiang District, 350005 Fuzhou, Fujian Province People’s Republic of China

**Keywords:** Obstructive sleep apnea, Hypoxia, Fatty liver, Liver injury

## Abstract

**Purpose:**

Obstructive sleep apnea (OSA) is suggested as a potential risk factor of nonalcoholic fatty liver disease (NAFLD). However, the underlying mechanism is still far from clear. The aim of this observational study was to investigate the influence of OSA-related hypoxia on severity of liver injury in patients with NAFLD.

**Methods:**

Consecutive patients with ultrasound-diagnosed NAFLD who underwent standard polysomnography were enrolled. Fasting blood samples were obtained from all patients for biological profile measurements, and demographic data were collected. Subjects were divided into control, moderate, and severe groups.

**Results:**

A total of 85 subjects with 73 males and 12 females were included (mean age, 44.67 ± 1.28 years; mean body mass index, 27.28 ± 0.33 kg/m^2^). Alanine aminotransferase (ALT), aspartate aminotransferase (AST), ALT/AST, gamma glutamyltransferase, total cholesterol, low density lipoprotein-cholesterol, fasting glucose, and high-sensitivity C-reactive protein significantly increased with the aggravation of OSA. In multivariate analysis, oxygen desaturation index was the major contributing factor for elevated ALT (*β* = 0.435, *p* = 0.000), average O_2_ saturation was the major independent predictor of elevated AST (*β* = −0.269, *p* = 0.020).

**Conclusions:**

OSA-related hypoxia was independently associated with the biochemical evidence of liver injury in the presence of NAFLD.

## Introduction

Obstructive sleep apnea (OSA) is characterized by repetitive episodes of partial or complete obstruction of the upper airway during sleep, resulting in sleep fragmentation and hypoxemia. The prevalence of OSA among the adult population is high, being 2 to 4 % in the general population and 35 to 45 % in obese individuals [1, 2]. There is accumulating evidence supporting the relationship of OSA with all manifestations of the metabolic syndrome, including visceral obesity, hypertension, dyslipidemia, and insulin resistance [3, 4]. An effective treatment for OSA has become available and continuous positive airway pressure (CPAP) may ameliorate metabolic outcomes [5, 6]. Recent data suggest that OSA is associated with another manifestation of metabolic dysfunction, nonalcoholic fatty liver disease (NAFLD) [7–9].

NAFLD represents a wide spectrum of liver disorders from isolated steatosis to nonalcoholic steatohepatitis (NASH) and cirrhosis. NAFLD is a common cause of chronic liver disease, affecting 30 % of the general adult population and up to 60 to 70 % of diabetic and obese patients [10]. Liver biopsy remains the gold standard to diagnose and stage NAFLD from steatosis to cirrhosis. Obesity, advanced age, diabetes mellitus, hypertriglyceridemia, and hypertension have been identified as the main risk factors of NAFLD [11]. A “two-hit” hypothesis has been proposed to explain the pathogenesis of NASH [12]. The “first hit” consists of excess hepatic triglyceride accumulation due to dysregulation of fatty acids and insulin resistance, in the absence of significant alcohol consumption or other liver disease. Whereas oxidative stress and cytokine expression constitute a “second hit”, leading to the shift from steatosis to NASH [11, 13].

In C57BL/6 J mice with diet-induced obesity (DIO), intermittent hypoxia for 6 months caused significant increase in serum transaminases, inflammation, and fibrosis. However, the DIO mice without hypoxia showed only hepatic steatosis but no histological evidence of steatohepatitis [14]. Given that chronic intermittent hypoxia (CIH) is a potential risk factor in the progression from fatty liver to NASH, we hypothesized that OSA-related hypoxia was independently associated with the biochemical evidence of liver injury in patients with ultrasound-diagnosed NAFLD.

## Materials and methods

### Subjects

**Fig. 1:**
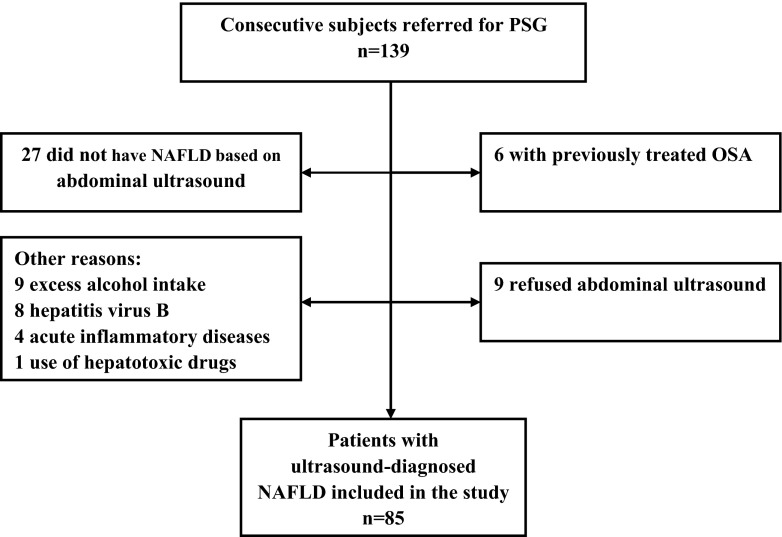
Study flow chart. Some subjects met more than one criterion for exclusion. *OSA* obstructive sleep apnea, *PSG* polysomnography, *NAFLD* nonalcoholic fatty liver disease

All consecutive patients who presented to our sleep laboratory because of symptoms of sleep apnea between December 2012 and October 2013 were included in the study. We selected subjects with ultrasound-diagnosed NAFLD. All patients completed an Epworth sleepiness scale (ESS) and a detailed questionnaire on sleep symptoms, history of alcohol consumption and smoking, medical history, and medications. Individuals with hepatitis virus B and/or C were excluded by systematic history taking and blood tests. Other exclusion criteria included known diagnosis of OSA and use of CPAP within the prior 3 months, excess alcohol intake (defined as >20 g/day for males and 10 g/day for females), current use of hepatotoxic drugs and other causes of chronic liver disease, severe cardiopulmonary chronic disease, or acute inflammatory disease. Figure 1 displays the study flow chart. Written informed consents were obtained from all subjects before the study, and this study had been approved by the local institutional ethics committee.

### Anthropometric and biochemical measurements

Body weight and height were measured in bare feet and light clothing in the morning with the same equipments. Body mass index (BMI) was calculated by dividing body weight to height square (kg/m^2^). Waist circumference was measured at a level midway between the lower costal margin and the iliac crest. Blood pressure was gauged by a standard mercury sphygmomanometer on the right arm with the participants in a sitting position after 5 min of rest—the average of two measurements, with 1-min interval, was considered. Fasting blood was taken in the morning for the measurement of serum glucose, alanine aminotransferase (ALT), aspartate aminotransferase (AST), alkaline phosphatase (Alk Phos), total bilirubin (T. bilirubin), gamma glutamyltransferase (GGT), and lipid profile comprising total cholesterol (TC), high-density lipoprotein-cholesterol (HDL-C), low-density lipoprotein-cholesterol (LDL-C), and triglycerides (TG). All blood samples were analyzed using the Modular P800 autoanalyzer (Roche, Tokyo, Japan). High-sensitivity C-reactive protein (hs-CRP) was measured with a BNII nephelometer (Dade Behring, Deerfield, IL, USA). The value of mean arterial pressure (MAP) was calculated by the following equation: diastolic blood pressure + 1/3 (systolic blood pressure − diastolic blood pressure).

### Polysomnographical evaluation

Overnight polysomnography (P Series Sleep System, Compumedics, Melbourne, Australia) was performed with recording of the following parameters: electroencephalography, electrooculography, electromyography, airflow using nasal and oral thermistors, respiratory effort using thoracic and abdominal impedance belts, arterial oxyhemoglobin saturation using pulse oximetry, snoring using tracheal microphone, and body position using a sensor. The polysomnography started, on average, at 22:00 hours (lights off) and finished around 06:00 hours (lights on). Sleep staging was scored according to the criteria of American Academy of Sleep Medicine (AASM) published in 2007 [15]. Apnea was defined as decrements in airflow ≥90 % from baseline for ≥10 s. Hypopnea was defined as a 30 % or greater decrease in flow lasting ≥10 s and was associated with a 4 % or greater oxyhemoglobin desaturation. The number of apneas and hypopneas per hour of sleep were calculated to obtain the apnea-hypopnea index (AHI). The oxygen desaturation index (ODI) was defined as the number of dips in oxygen saturation (SpO_2_) ≥4 % per hour of total sleep time. Polysomnographical parameters including lowest O_2_ saturation (LaSO_2_), mean nocturnal oxygen saturation (average SpO_2_), and the percentage of sleep time with SpO_2_ < 90 % (T90 %) were also recorded. OSA was defined as absent when AHI was <10 events/h, moderate when it was 10–50 events/h, and severe when it was ≥50 events/h [16].

### Definition of NAFLD

**Fig. 2:**
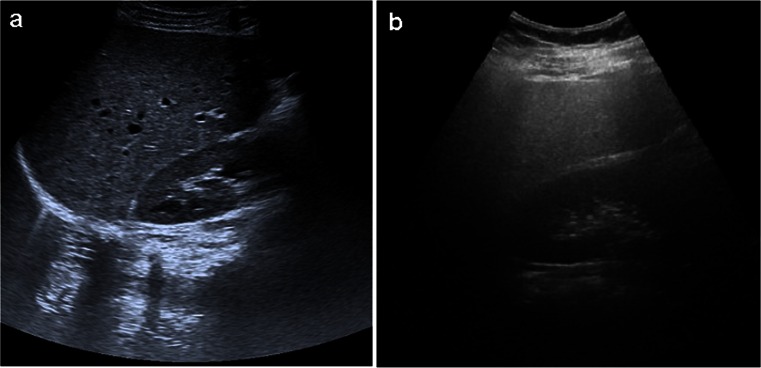
Abdominal US of a patient without NAFLD (**a**) in comparison with a patient with NAFLD (**b**). US scan of the liver of a 26-year-old patient without NAFLD showed solid homogenous echo texture (**a**). US scan of the liver of a 45-year-old patient with NAFLD showed diffusely increased liver echogenicity, blurring of vascular, and increased acoustic attenuation (**b**). *US* ultrasonography

Abdominal ultrasound was performed using a 3.5 MHz transducer (Technos DU 8, Genoa, Italy) by an experienced radiologist who was unaware of the aim of the study and blinded to the laboratory values. Both subcostal and intercostal scanning was done. Images were captured in a standard fashion with the subject in the supine position and with the right arm raised above the head. Normal liver parenchyma was seen as solid homogenous echo texture, which was midway between the renal cortex and pancreatic echogenicity (Fig. 2a). Fatty liver disease was diagnosed as diffusely increased echogenicity of the hepatic parenchyma compared to the kidneys, vascular blurring, and deep-echo attenuation [17, 18] (Fig. 2b). NAFLD was defined as subjects with fatty liver disease and no history of excessive alcohol consumption.

### Statistical analysis

Statistical analyses were performed using SPSS v 17.0 (SPSS Inc., Chicago, IL, USA). All variables were tested for normal distribution before analysis. Data were expressed as mean ± SD, median (interquartile range), and number (percentage) for normally distributed, skewed, and categorical data, respectively. Normally distributed continuous variables were compared by using one-way ANOVA for multiple-group comparison. Non-normally distributed continuous variables were compared by using Kruskal–Wallis H (K) for multiple-group comparison. Chi-square test or Fisher’s exact test was performed for categorical variables. Correlations between variables were explored using the Spearman rank test. All descriptive data not in normal distribution were ln-transformed before multivariate analysis. Multiple linear regression analysis was performed to determine the independent predictors of ALT and AST. A stepwise selection method was used in the multivariate analysis. Differences were considered significant if the *p* value was <0.05.

## Results

Eighty-five subjects with ultrasound-diagnosed NAFLD, including 73 males and 12 females, were finally included (Fig. 1). The mean BMI was 27.28 ± 0.33 kg/m^2^ (range, 21.05–35.59 kg/m^2^) and mean age was 44.67 ± 1.28 years (range, 20–72 years). OSA (AHI ≥10 events/h) found in 71 (83.5 %) patients, was moderate in 36 patients, and severe (AHI ≥50 events/h) in 35 patients.

The anthropometric and polysomnographic characteristics of the patients are presented in Table 1. Polysomnographical parameters including LaSO_2_, average SpO_2_ decreased significantly with an increase in OSA severity, whereas BMI, neck circumference, waist circumference, AHI, ODI, and T90 % increased with OSA severity (all *p* = 0.000). There were no significant differences among the three groups for age, current smoking, sex ratio, medications, or ESS score.

**Table 1:** Anthropometric and polysomnographic parameters of all patients according to the presence of OSA and its severity

	Controls	Moderate OSA	Severe OSA	*p* values
Subjects *n*	14	36	35	
Age, years	46.21 ± 11.81	44.14 ± 8.95	44.60 ± 14.29	0.857
Male sex, number (%)	11 (78.6)	32 (88.9)	30 (85.7)	0.592
Antihypertensive agents, number (%)	3 (21.4)	8 (22.2)	6 (17.1)	0.878
Antidiabetic agents, number (%)	2 (14.3)	1 (2.8)	1 (2.9)	0.275
Antilipemic agents, number (%)	0 (0.0)	0 (0.0)	1 (2.9)	0.576
Current smoking, number (%)	5 (35.7)	10 (27.8)	11 (31.4)	0.853
Body mass index (kg/m^2^)	24.85 ± 2.52	26.86 ± 2.70	28.69 ± 2.80	0.000
Neck circumference (cm)	37.29 ± 2.77	39.68 ± 2.96	42.00 ± 3.18	0.000
Waist circumference (cm)	90.25 ± 5.13	97.19 ± 7.72	102.17 ± 6.95	0.000
AHI	5.00 (3.48–6.90)	24.10 (17.50–37.00)	64.10 (58.10–76.40)	0.000
ODI	2.60 (1.58–8.33)	19.47 (11.93–29.85)	60.40 (49.81–73.75)	0.000
T90 %(%)	0.10 (0.01–0.32)	3.14 (0.70–6.12)	20.04 (9.60–44.58)	0.000
LaSO_2_ (%)	87.00 (82.75–89.25)	75.00 (68.25–84.00)	66.00 (49.00–71.00)	0.000
Average SpO_2_ (%)	96.00 (94.00–96.25)	94.00 (93.00–95.00)	90.00 (85.00–92.00)	0.000
ESS score	9.07 ± 5.08	9.06 ± 4.80	11.60 ± 5.49	0.087

Normally distributed data were expressed as mean ± SD, skewed data (including AHI, ODI, T90 %, LaSO_2_, average SpO_2_) were presented as median (interquartile range). Categorical variables were expressed as number (percentage)

*OSA* obstructive sleep apnea, *AHI* apnea-hypopnea index, *ODI* oxygen desaturation index, *T90* % the percentage of total sleep time spent with SpO_2_ < 90 %, *LaSO*_*2*_ lowest O_2_ saturation, *average SpO*_*2*_ average O_2_ saturation, *ESS score* Epworth Sleepiness Scale score

The metabolic and liver function parameters are reported in Table 2. The three groups did not differ in terms of MAP, TG, T. bilirubin, or Alk Phos. A strong positive association was observed between OSA severity and the indices of TC, LDL-C, hs-CRP, GGT, and ALT/AST (*p* = 0.001, *p* = 0.005, *p* = 0.000, *p* = 0.000, and *p* = 0.001, respectively). In addition, a statistically significant positive association with the fasting glucose as well as negative association with HDL-C was seen. ALT increased from 19.00 to 54.00 U/L with OSA severity (*p* = 0.000). AST increased from 21.50 to 36.00 U/L with OSA severity (*p* = 0.001). A statistically significant increase in the prevalence of elevated serum aminotransferases was observed from controls to severe OSA group.

**Table 2:** The metabolic and liver function parameters of all patients according to the presence of OSA and its severity

	Controls	Moderate OSA	Severe OSA	*p* values
Subjects *n*	14	36	35	
MAP (mmHg)	93.17 ± 8.93	94.99 ± 7.95	98.67 ± 13.18	0.177
TC (mmol/L)	4.88 ± 0.91	4.94 ± 0.80	5.65 ± 0.92	0.001
TG (mmol/L)	1.69 (1.37–3.09)	1.93 (1.40–2.70)	2.21 (1.46–3.38)	0.455
HDL-C (mmol/L)	1.26 ± 0.45	1.03 ± 0.20	1.09 ± 0.24	0.036
LDL-C (mmol/L)	2.95 ± 0.83	3.12 ± 0.80	3.66 ± 0.81	0.005
Fasting glucose (mmol/L)	5.06 (4.40–6.44)	5.37 (4.98–5.77)	5.98 (5.28–6.79)	0.028
Hs-CRP (mg/L)	0.48 (0.25–1.04)	0.99 (0.55–2.79)	3.26 (1.77–4.59)	0.000
ALT (U/L)	19.00 (16.50–26.00)	31.50 (26.00–48.50)	54.00 (31.00–81.00)	0.000
AST (U/L)	21.50 (18.75–24.00)	25.00 (20.25–31.75)	36.00 (23.00–49.00)	0.001
ALT/AST ratio	1.00 ± 0.36	1.35 ± 0.37	1.54 ± 0.51	0.001
Elevated ALT^a^, number (%)	1 (7.1)	13 (36.1)	22 (62.9)	0.001
Elevated AST^a^, number (%)	0 (0.0)	4 (11.1)	13 (37.1)	0.003
T. bilirubin (umol/L)	11.13 ± 4.40	10.32 ± 3.75	12.33 ± 4.40	0.129
Alk Phos (U/L)	69.43 ± 16.12	75.06 ± 25.29	71.54 ± 14.58	0.613
GGT (U/L)	21.00 (19.75–29.50)	38.00 (26.00–69.00)	67.00 (40.00–95.00)	0.000

Normally distributed data were expressed as mean ± SD, skewed data (including TG, fasting glucose, hs-CRP, ALT, AST, and GGT) were presented as median (interquartile range). Categorical variables were expressed as number (percentage)

*OSA* obstructive sleep apnea, *MAP* mean arterial pressure, *TC* total cholesterol, *TG* triglycerides, *HDL*-*C* high-density lipoprotein-cholesterol, *LDL*-*C* low-density lipoprotein-cholesterol, *Hs*-*CRP* high-sensitivity C-reactive protein, *ALT* alanine aminotransferase, *AST* aspartate aminotransferase, *T. bilirubin* total bilirubin, *Alk Phos* alkaline phosphatase, *GGT* gamma glutamyltransferase

^a^Elevated ALT and AST were defined as higher than the upper limit of normal

**Fig. 3:**
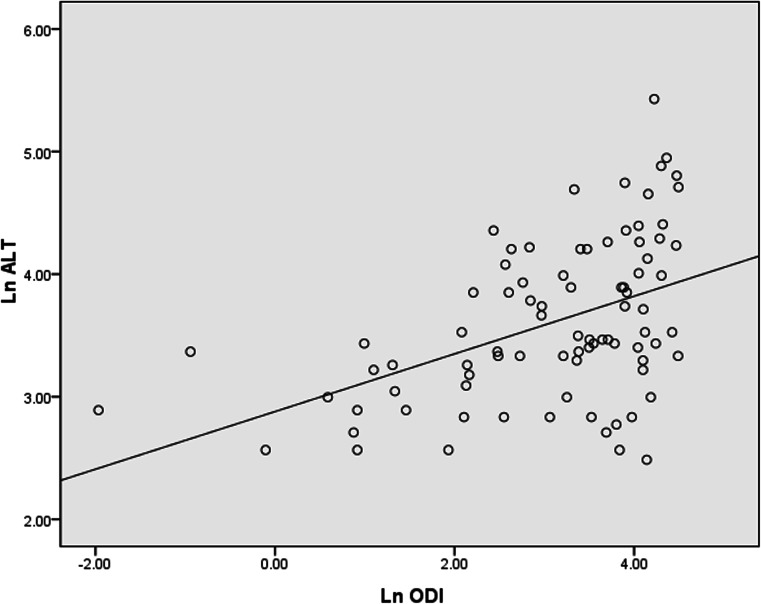
Correlation between ln ALT and ln ODI. Age, gender, current smoking, BMI, waist circumference, MAP, LDL-C, HDL-C, TC, ln TG, ln fasting glucose, ln hs-CRP, ln AHI, ln ODI, ln T90 %, ln LaSO_2_, and ln average SpO_2_, as independent variables, were entered into the regression model, and ln ALT was taken as dependent variable; stepwise linear regression showed that age, ln TG, and ln ODI were included in the final model. Ln ODI was the independent predictor of ln ALT (*β* = 0.435, adjusted *r* ^2^ = 0.322, *p* = 0.000). *BMI* body mass index, *MAP* mean arterial pressure, *LDL*-*C* low-density lipoprotein-cholesterol, *HDL*-*C* high-density lipoprotein-cholesterol, *TC* total cholesterol, *TG* triglycerides, *Hs*-*CRP* high-sensitivity C-reactive protein, *AHI* apnea-hypopnea index, *ODI* oxygen desaturation index, *T90* % the percentage of total sleep time spent with SpO_2_ < 90 %, *LaSO*_*2*_ lowest O_2_ saturation, *average SpO*_*2*_ average O_2_ saturation, *ALT* alanine aminotransferase

**Fig. 4:**
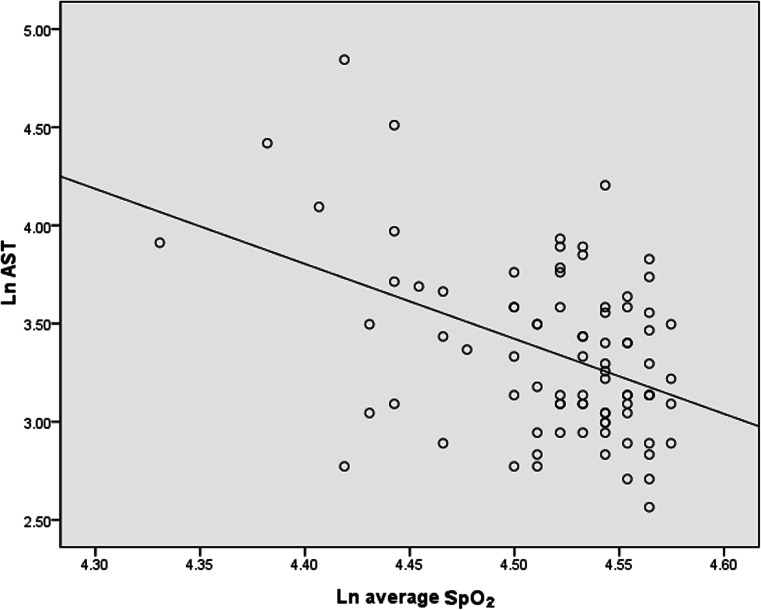
Correlation between ln AST and ln average SpO_2_. Age, gender, current smoking, BMI, waist circumference, MAP, LDL-C, HDL-C, TC, ln TG, ln fasting glucose, ln hs-CRP, ln AHI, ln ODI, ln T90 %, ln LaSO_2_, and ln average SpO_2_, as independent variables, were entered into the regression model, and ln AST was taken as dependent variable; stepwise linear regression showed that ln average SpO_2_, ln TG, and ln AHI were included in the final model. Ln average SpO_2_ was the independent predictor of ln AST (*β* = −0.269, adjusted *r* ^2^ = 0.244, *p* = 0.020). *BMI* body mass index, *MAP* mean arterial pressure, *LDL*-*C* low-density lipoprotein-cholesterol, *HDL*-*C* high-density lipoprotein-cholesterol, *TC* total cholesterol, *TG* triglycerides, *Hs*-*CRP* high-sensitivity C-reactive protein, *AHI* apnea-hypopnea index, *ODI* oxygen desaturation index, *T90* % the percentage of total sleep time spent with SpO_2_ < 90 %, *LaSO*_*2*_ lowest O_2_ saturation, *average SpO*_*2*_ average O_2_ saturation, *AST* aspartate aminotransferase

Table 3 shows the correlations between ALT, AST, and the other variables. Both indices were significantly correlated with BMI, waist circumference, fasting glucose, TG, TC, AHI, ODI, T90 %, and average SpO_2_ (all *p* < 0.05). Hs-CRP, as an expression of inflammation, was also correlated with ALT and AST (*r* = 0.263, *p* = 0.015 and *r* = 0.349, *p* = 0.001, respectively). Stepwise multiple linear regression analyses were used to determine predictors of ALT and AST. These analyses identified ln ODI (*β* = 0.435, *p* = 0.000), ln TG (*β* = 0.259, *p* = 0.008) and age (*β* = −0.190, *p* = 0.046) as independent explanatory variables for ln ALT (Fig. 3), ln average SpO_2_ (*β* = −0.269, *p* = 0.020), ln TG (*β* = 0.221, *p* = 0.024) and ln AHI (*β* = 0.226, *p* = 0.047) for ln AST (Fig. 4).

**Table 3:** Spearman rank correlation coefficients between liver enzyme levels (ALT and AST) and anthropometric, metabolic, and polysomnographic characteristics

	ALT		AST	
	*r*	*p* values	*r*	*p* values
Age	−0.264	0.015	−0.099	0.366
Body mass index	0.335	0.002	0.347	0.001
Waist circumference	0.342	0.001	0.372	0.000
AHI	0.512	0.000	0.469	0.000
ODI	0.485	0.000	0.444	0.000
T90 %	0.330	0.002	0.300	0.005
Average SpO_2_	−0.304	0.005	−0.296	0.006
LaSO_2_	−0.235	0.030	−0.206	0.059
MAP	0.091	0.407	0.119	0.277
Fasting glucose	0.247	0.023	0.321	0.003
TC	0.215	0.048	0.214	0.049
TG	0.333	0.002	0.241	0.026
HDL-C	−0.172	0.116	−0.034	0.754
LDL-C	0.101	0.358	0.122	0.268
Hs-CRP	0.263	0.015	0.349	0.001

*ALT* alanine aminotransferase, *AST* aspartate aminotransferase, *AHI* apnea-hypopnea index, *ODI* oxygen desaturation index, *T90* % the percentage of total sleep time spent with SpO_2_ < 90 %, *average SpO*_*2*_ average O_2_ saturation, *LaSO*_*2*_ lowest O_2_ saturation, *MAP* mean arterial pressure, *TC* total cholesterol, *TG* triglycerides, *HDL*-*C* high-density lipoprotein-cholesterol, *LDL*-*C* low density lipoprotein-cholesterol, *Hs*-*CRP* high-sensitivity C-reactive protein

## Discussion

In this cross-sectional study performed in a group of ultrasound-diagnosed NAFLD patients with both sexes, we found that OSA commonly affected patients with NAFLD (83.5 %). Nocturnal hypoxia was associated with the elevation of liver enzymes, independent of a variety of relevant factors such as age, gender, obesity, inflammation, blood pressure, serum glucose, and lipid profile, which suggested that nocturnal hypoxia might be a risk factor in the progression of NAFLD.

Several studies evaluating the relationship between OSA and NAFLD have been conducted, and the results were conflicting. A study including 109 OSA patients showed that ALT and AST levels significantly correlated with the severity of nocturnal hypoxia, but not with the AHI or metabolic syndrome features. On regression analysis, T90 % remained a stronger predictor of the variance in ALT [19]. It should be noted that this study did not include a control group. Kheirandish-Gozal and colleagues [20] reported significantly higher prevalence of elevated ALT levels in children with OSA compared to children without OSA. Mishra et al. [8] studied 101 bariatric patients with biopsy-proven NAFLD, all of whom had full PSG in a sleep laboratory. Subjects with histological NASH had significantly lower LaSO_2_, lower average SpO_2_, higher AHI, and higher ALT/AST compared with non-NASH controls. Multivariate analysis showed that the LaSO_2_ was independently associated with histological NASH. Polotsky et al. [9] studied 90 consecutive bariatric patients who underwent PSG, and reported that nocturnal oxygen desaturation might predispose to hepatic inflammation, hepatocyte ballooning, and liver fibrosis. Notably, ALT and AST values were within the normal range in all patients. This study was limited by the fact that the number of subjects from whom liver biopsies were available was small. More recently, a study based on pediatric patients with liver biopsy-proven NAFLD identified that the severity and duration of hypoxemia were associated with both histological measures of NAFLD disease severity and with elevated AST and ALT levels [21]. However, Turkay and co-workers [22] found that there were no significant differences in the results of liver function tests grouped by severity of obstructive sleep apnea. Singh et al. [23] evaluated 190 patients with a biochemical diagnosis of NAFLD, of whom 50 had undergone hepatic biopsy. They did not find a difference between the prevalence of OSA symptoms in the patients with steatosis and steatohepatitis. Another study which enrolled 40 bariatric patients also elucidated that there was no association between AHI or oxyhemoglobin desaturation and liver enzymes, hepatic histologic features, or NASH overall [24]. Factors such as sample size, population, diagnosis methodology, and study design may be responsible for the discrepancies in study outcomes.

We focused on patients with ultrasound-diagnosed NAFLD and demonstrated that ODI and average SpO_2_ were the determinant factors predicted serum liver enzyme levels after controlling for confounders. At the same time, our results showed that liver injury was better predicted by markers of oxygen desaturation than by AHI, which was consistent with previous reports [21, 7, 9]. The findings of our study suggest that in the setting of NAFLD, repeated nocturnal hypoxemic episodes may act as the “second hit” leading to the progression of NAFLD. A recently published study including a large range of BMI patients from lean to overweight and massively obese demonstrated a dose-response relationship between the severity of nocturnal hypoxia and liver injury only in morbidly obese but not in lean [25]. This hypothesis has been further supported by animal experiments. In diet-induced obesity models, mice exposed to CIH exhibited elevated aminotransferase levels, hepatic steatosis, lobular inflammation, and fibrosis, while mice exposed to intermittent air showed only hepatic steatosis. The changes in mice exposed to CIH were coupled with significant increases in serum and liver lipid peroxidation products and in hepatic myeloperoxidase, proinflammatory cytokines, chemokine macrophage inflammatory protein-2, and α1 (I)-collagen expression [14]. In another experiment, C57BL/6 J mice were fed a high fat diet for 12 weeks and then exposed to CIH or control conditions for 4 weeks. CIH doubled HOMA index, induced severe glucose intolerance, significantly increased liver enzymes, inflammation, and oxidative stress [26].

Conflicting results were also observed in the effect of CPAP treatment on NAFLD. Chin et al. [27] observed that treatment with CPAP produced a significant reduction in aminotransferases during the first night of treatment; these improvements were maintained after 1 and 6 months of nasal CPAP treatment. A significant improvement in liver enzymes after effective treatment of OSA has also been demonstrated in obese kids [20]. Conversely, a randomized placebo-controlled study failed to show this beneficial effect on liver enzymes in patients with moderate-to-severe OSA after 4 weeks of treatment with CPAP compared to sham CPAP [28]. Taken together, there is clearly a need for large-scale, randomized controlled interventional trials to assess whether effective versus sham CPAP treatment is able to improve liver injury.

There are several limitations of our study that will require further evaluation. Its cross-sectional design allowed us to support a correlation but not a causative relationship between OSA and the severity of NAFLD. Second, the sample size of the present study was relatively small, and it could not be analyzed separately by sex since female subjects were few. In addition, we excluded other rare forms of chronic liver disease such as Wilson disease, *α*1-anti-trypsin deficiency, and auto-immune hepatitis by medical history rather than blood test. Furthermore, confounding factors such as BMI and waist circumference were not matched for each group, and multivariate analysis was performed to overcome the limitation. Another potential limitation is the use of serum aminotransferases as a surrogate marker of liver injury. Various other noninvasive methods to assess NAFLD have been used, including Steato Test for hepatic steatosis, Nash Test for NASH, and Fibro Test for liver fibrosis. Although liver biopsy is the best way to confirm the diagnosis of NAFLD and to determine the severity of this disease, it could not be widely performed for ethical reasons. However, previous studies have reported less than 10 % of nonalcoholic fatty liver and as many as 100 % of NASH patients with elevated serum aminotransferases [29, 30], and this method has been employed previously by a number of studies to examine potential links between OSA and NAFLD [23, 19, 27].

In conclusion, our study showed that nocturnal hypoxia in patients with OSA was a risk factor of liver injury in the presence of NAFLD. For a better understanding of this complex relationship, further randomized controlled studies including patients with histologically proven NAFLD and PSG defined OSA are needed to establish whether there is an independent association between them.
